# Pathogenesis of Congenital Rubella Virus Infection in Human Fetuses: Viral Infection in the Ciliary Body Could Play an Important Role in Cataractogenesis

**DOI:** 10.1016/j.ebiom.2014.10.021

**Published:** 2014-10-30

**Authors:** Thong Van Nguyen, Van Hung Pham, Kenji Abe

**Affiliations:** a Department of Pathology, Cytology and Genetics, Hung Vuong Hospital, Ho Chi Minh City, Viet Nam; b Center for Molecular Biomedicine, School of Medicine, University of Medicine and Pharmacy in Ho Chi Minh City, Ho Chi Minh City, Viet Nam; c Molecular Diagnostics Section, Nam Khoa-Biotek Laboratory, Ho Chi Minh City, Viet Nam; d Department of Pathology, National Institute of Infectious Diseases, Tokyo, Japan

**Keywords:** Pathology of rubella, Pathogenesis of rubella virus, Congenital rubella infection (CRI), Congenital rubella syndrome (CRS), Cataract

## Abstract

**Background:**

Development of congenital rubella syndrome associated with rubella virus infection during pregnancy is clinically important, but the pathogenicity of the virus remains unclear.

**Methods:**

Pathological examination was conducted on 3 aborted fetuses with congenital rubella infection.

**Findings:**

At autopsy, all 3 aborted fetuses showed congenital cataract confirmed by gross observation. Rubella virus infection occurred via systemic organs including circulating hematopoietic stem cells confirmed by immunohistochemical and molecular investigations, and major histopathogical changes were found in the liver. It is noteworthy that the virus infected the ciliary body of the eye, suggesting a possible cause of cataracts.

**Interpretation:**

Our study based on the pathological examination demonstrated that the rubella virus infection occurred via systemic organs of human fetuses. This fact was confirmed by immunohistochemistry and direct detection of viral RNA in multiple organs. To the best of our knowledge, this study is the first report demonstrating that the rubella virus infection occurred via systemic organs of the human body. Importantly, virus infection of the ciliary body could play an important role in cataractogenesis.

## Highlights

• This study demonstrates that congenital rubella virus infection occurred via systemic organs of human fetuses.
• Virus infection of the ciliary body could play an important role in cataractogenesis.

## 1 Introduction

Rubella disease is now prevented by vaccines, but remains poorly controlled in developing countries including Southeast Asia. Rubella is an acute infectious disease that normally follows a mild clinical course. However, infections during pregnancy, especially before week 12 of gestation, can cause severe birth defects known as congenital rubella syndrome (CRS) (Banatvala and Brown, 2004, Duszak, 2009). Clinical signs of CRS include cataract, glaucoma, heart disease, loss of hearing, brain dysfunction, and pigmentary retinopathy. These illnesses are clinically important, yet the pathogenesis of rubella virus (RV) infection in fetuses/newborns remains obscure due to the lack of a suitable animal model for this purpose. There have been very few reports in the literature of histopathological studies on CRS in humans and most appeared in the period from late 1960 to early 1970 (Töndury and Smith, 1966, Brookhouser and Bordley, 1973, Menser and Reye, 1974).

In Vietnam, rubella epidemics occurred during the period from 2011 to 2012. Through this outbreak, our investigation based on molecular epidemiology showed that RV RNA was detectable in the placenta from all of 10 aborted fetuses and 10 newborns from pregnant women with rubella (Pham et al., 2013). Importantly, all newborns and aborted fetuses were found by gross examination to have congenital cataracts. To obtain more detailed information, we conducted further histopathological and immunohistochemical examinations in the aborted fetuses and herein discuss the pathogenicity of RV infection in human fetuses.

## 2 Patients and Methods

### 2.1 Patients

We examined 3 aborted fetuses. All mothers of the 3 aborted fetuses had a history of rubella with a rash, fever and lymph node swelling at weeks 5–6 of gestation. Ultrasound imaging in these mothers showed hyperechogenic lesions in the liver, kidney and bowel of all fetuses. Abortion was carried out at weeks 23, 22 and 13 of gestation, respectively.

### 2.2 Ethical Approval

This study conforms to the ethical guidelines and was approved by the ethics committees of the Hung Vuong Hospital, Ho Chi Minh City, Vietnam. Written informed consent in this study was obtained from all of the mothers.

### 2.3 Histopathological and Immunohistochemical Examination

Tissue samples obtained at the autopsy were divided into two portions. One portion was used for conventional histological examination and the other for viral RNA detection. In the former, tissue samples were fixed in 10% buffered formalin and embedded in paraffin blocks for histopathological and immunohistochemical examinations. In the latter, tissue samples were frozen and stored at − 80 °C until use.

To examine distribution of RV-related antigen in multiple organs, thin sections of formalin-fixed paraffin-embedded tissues were stained immunohistochemically by an avidin–biotin complex immunoperoxidase method (LSAB2 kit/HRP/DAB; Dako Cytomation, Copenhagen, Denmark) using a mouse monoclonal antibody against RV capsid protein (Abcam Ltd., Cambridge, UK). Furthermore, to identify hematopoietic stem cells in tissues, human CD34 antigen was immunostained using a mouse monoclonal antibody (CD34 Class II, Dako Cytomation). Normal mouse serum as the primary antibody was used for the negative control.

### 2.4 Detection of RV Gene by Nested Reverse-transcriptase (RT)-PCR

Total RNA was extracted from frozen tissue specimens using the RNA extraction kit (^NK^RNAPREP kit, Nam Khoa Biotek Co., Ho Chi Minh City, Vietnam). Viral cDNA was synthesized with mixture of random primer and oligo(dT) primer using iScript reverse transcriptase (Bio-Rad Laboratories, CA, USA) with the following condition: 25 °C, 5 min, 42 °C, 30 min and 85 °C, 5 min. For RV gene amplification, hemi-nested PCR was carried out with primers designed from the E1 gene of RV. RV gene fragment was amplified by the primer combination of RV8537F (5′-GGG TAC GCG CAG CTG GCG TC-3′; sense, nt 8537–8556) and RV9117R (5-CAY TTG CGC GCC TGM GAG CC-3′; antisense, nt 9117–9098) for the outer primer pairs (581 bp) and RV8633F (5′-AGC GAC GCR GCS TGC TGG GG-3′; sense, nt 8633–8652) and RV9117R for the inner primer pairs (485 bp). Nucleotide position is based on rubella virus vaccine strain wistar RA 27/3 (accession # FJ211587).

Five μl of cDNA product was placed in PCR buffer containing Platinum Taq and 360 GC Enhancer (20% v/v; Applied Biosystems, Foster City, CA, USA) due to the RV genome having an extremely high GC-rich sequence. Amplification conditions included pre-incubation at 95 °C, 5 min, followed by 40 cycles consisting of 94 °C, 30 s, 60 °C, 30 s and 72 °C, 1 min for the 1st round PCR and 94 °C, 30 s, 65 °C, 30 s and 72 °C, 1 min for the 2nd round PCR.

In addition, we also examined to detect negative-strand RV RNA, which indicates the replicative form of the positive-strand RNA virus in infected cells. To detect the negative-strand RV RNA, viral cDNA was synthesized with RV-specific sense primer (RV8537F) using iScript reverse transcriptase with the following condition: 25 °C, 5 min, 42 °C, 30 min and 95 °C, 5 min. Obtained viral cDNA was amplified by the hemi-nested PCR with the same condition as described above.

Amplicons were analyzed by electrophoresis on 2% agarose gels staining with ethidium bromide and recovered using the Promega Wizard® SV Gel and PCR Clean-Up System (Promega, Madison, WI, USA). The amplicons were subjected to direct sequencing using the ABI PRISM™ Big Dye Terminator Cycle Sequencing Ready Reaction Kit (Applied Biosystems), on a capillary sequencer model 3130 (Applied Biosystems).

## 3 Results

**Fig. 1:**
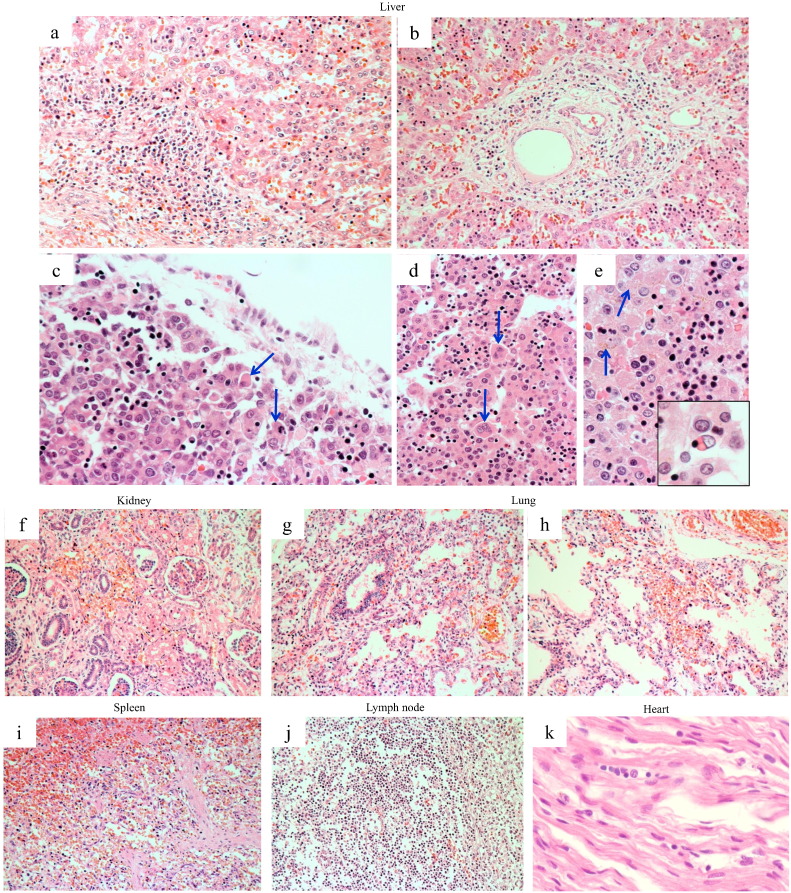
Histopathological findings of multiple organs from RV-infected fetuses; the liver shows congestion, expansion of the portal area with inflammatory infiltrate and piecemeal necrosis, vacuolar degeneration of epithelial cells in the intrahepatic bile ducts (a, b), necrotizing and inflammatory changes with apoptotic hepatocytes (so called acidophil bodies; arrows) (c), giant cell formation of hepatocytes (arrows) (d), mild deposition of bile pigments into the hepatocytes (arrows) (e) and erythrophagocytosis by Kupffer cell (inset of e). Mild nephritis with partial hemorrhage in the kidney (f), minor pneumonia accompanied by congestion, alveolar hemorrhage and interstitial edema in the lung (g, h), hypoplasia with hemorrhage in the spleen (i) and lymph node (j), mild myocarditis with interstitial edema in the heart (k) can be seen. H&E stain.

At autopsy, all 3 aborted fetuses showed a congenital cataract confirmed by gross observation. Histologically, the following findings were observed. In the liver, congestion, necrotizing and inflammatory changes accompanied with hemorrhage, apoptotic hepatocytes (so called acidophil bodies), expansion of the portal area with mild to moderate inflammatory infiltrate (rarely with piecemeal necrosis), giant cell formation of hepatocytes, mild deposition of bile pigments into the hepatocytes and vacuolar degeneration of epithelial cells in the intrahepatic bile ducts were observed (Fig. 1a–d). Interestingly, active erythrophagocytosis by Kupffer cells suggesting virus-associated hemophagocytic syndrome was seen (Fig. 1e). The kidney showed mild nephritis accompanied by partial hemorrhage (Fig. 1f). In the lung, all cases had mild pneumonia accompanied by congestion, alveolar hemorrhage and interstitial edema (Fig. 1g, h). The spleen and lymph node from one case showed hypoplasia with hemorrhage (Fig. 1i, j). The heart exhibited mild myocarditis accompanied by interstitial edema (Fig. 1k). In the central nervous system (CNS), no remarkable change suggesting viral encephalitis was found.

**Fig. 2:**
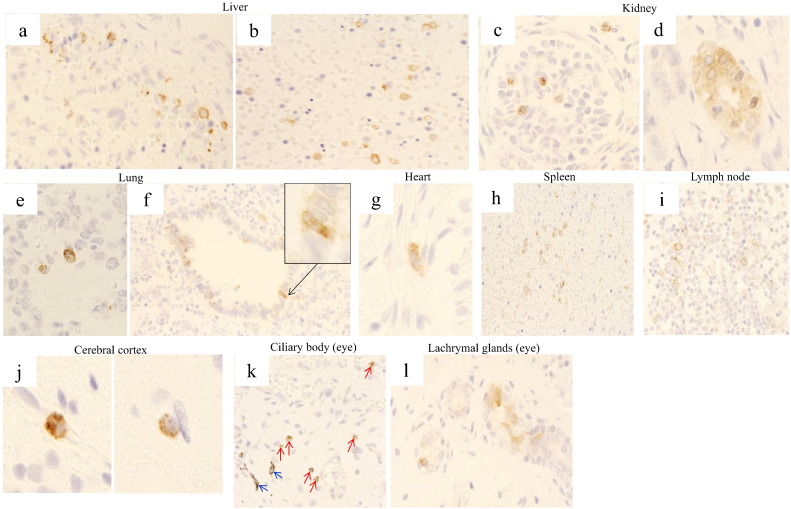
Immunohistochemical staining shows distribution of RV capsid antigen in multiple organs. Positive reaction of RV antigen on the surface of hematopoietic mononuclear cells produced in the fetal liver (a, b). Localization of the RV antigen in the epithelial cells of the glomerulus (c) and proximal tubules (d) in the kidney, the epithelial cells of the alveolus (e) and bronchioles (f; inset shows a higher magnification) in the lungs, myocardial cells in the heart (g), spleen cells (h), lymphoid tissue (i), and nerve cells in the cerebral cortex (j). Importantly in the eye, virus antigen was detected in the epithelial cells of the ciliary body (k; red arrows indicates virus antigen and blue arrows indicates melanin pigment cells) and the lachrymal glands (l). The RV capsid antigen is prominent in the cytoplasm of all infected cells. See the text for a more detailed description of these findings.

The immunohistochemical examination revealed localization of RV capsid antigen in multiple organs from all 3 cases tested (Table 1). In the liver, which showed necrotizing and inflammatory changes, virus antigen was localized on the surface of hematopoietic mononuclear cells produced in the fetal liver (Fig. 2a, b). These mononuclear cells tested positive for cell surface marker CD34 by immunohistochemical examination, suggesting that they were derived from hematopoietic stem cells. Similar RV infected hematopoietic stem cells expressing CD34 were also observed in the systemic organs including the spleen, kidney, lungs, heart, CNS and eyes.

Virus antigen was also localized in the epithelial cells of the glomerulus and proximal tubules of the kidney, bronchioles and alveolus in the lungs, myocardial cells in the heart, spleen cells, lymphoid tissue, and nerve cells in the cerebral cortex (Fig. 2c–j). Furthermore, an important finding in the eye was the presence of virus antigen in the epithelial cells of the ciliary body and the lachrymal glands (Fig. 2k, l). RV capsid antigen was prominent in the cytoplasm of all infected cells. No positive reactions using normal mouse serum as the negative control were seen.

By the nested RT-PCR assay using tissues from multiple organs, positive-strand RV RNA was detectable in nasal swabs, placenta and the lens of the eye in all 3 cases tested. Furthermore, viral RNA was also detectable in all of the major organs including the liver, kidney, spleen, heart, lungs, the eye and CNS (consisting of the cerebral cortex, cerebellum and brain stem) obtained from all fetuses examined (Table 1). In addition, our examination detected negative-strand RV RNA, which indicates the replicative form of the positive-strand RNA virus in infected cells. This result showed that all samples tested were positive for the negative-strand RNA of the virus.

To confirm the sequence identity of the virus genes, amplicons detected in liver tissues from 3 cases were sequenced. The result showed that nucleotide sequences of RV from the liver tissues were identical among all 3 cases. Furthermore, these 3 strains isolated in the tissues also showed identical nucleotide sequences to those of RV strains identified in the rubella outbreak season during the period from 2011 to 2012.

## 4 Discussion

Clinically, the most important illness during congenital rubella infection is the development of malformations in the fetus (Banatvala and Brown, 2004, Duszak, 2009). In particular, it is known that the fetus of early pregnancy is at greater risk than at later stages. So far, many epidemiological studies on RV infection have been reported from many countries, but there are very few reports of pathological studies on rubella (Töndury and Smith, 1966, Brookhouser and Bordley, 1973, Menser and Reye, 1974). For this reason, the pathogenesis of RV infection in human fetuses remains unknown. In addition, the lack of appropriate animal models for rubella infection impedes efforts to elucidate this issue.

In this study, we analyzed the pathological features of RV infection in human fetuses having CRS. Our results presented here indicate that the route of RV infection was via the systemic organs of the human fetuses. This fact has been confirmed by immunohistochemistry and direct detection of viral RNA in multiple organs. This was also demonstrated by the detection of negative-stranded RNA of RV, which indicates the replicative form of positive-stranded RNA virus in infected cells.

Major histopathological change was observed in the liver. The liver of the embryo plays a very important role in hematopoiesis instead of bone marrow. In the liver from infected fetuses, necrotizing and inflammatory changes suggesting viral hepatitis were present. However, obvious inflammatory changes caused by virus infection were mild in other organs from infected fetuses. In the CNS, no remarkable change suggesting viral encephalitis was found despite the virus infected the nerve cells in the cerebral cortex. This reason is not clear, but as one of the reasons, it might be due to differences in the response to the virus in the embryonic brain.

The finding of viral antigen localized in epithelial cells of the glomerulus and proximal tubules in the kidney suggests excretion of the virus in the urine. In fact, RV can be detected in the urine from rubella patients. Furthermore, RV antigen can be detected frequently in mononuclear cells from multiple organs, and RV is distributed to the whole body through circulating infected mononuclear cells. In fetal life, most of the mononuclear cells express CD34 antigen suggesting that they are hematopoietic stem cells produced mainly in the liver.

One of the important illnesses related to CRS is cataract. In our previous study in Vietnamese patients, all of 20 fetuses/newborns with congenital RV infection presented with congenital cataract (Pham et al., 2013). In the pathological study reported here, we obtained direct evidence of viral infection in the epithelial cells of the ciliary body and the lachrymal glands in the eye. Physiological function of the ciliary body is the production of aqueous humor (Goel et al., 2010). The aqueous humor is analogous to a blood surrogate for these avascular structures and provides oxygen and nutrition to the lens, removes excretory products of metabolism, transports neurotransmitters, stabilizes the ocular structure and contributes to the regulation of the homeostasis of these ocular tissues. This cycle is called the aqueous circulation (Goel et al., 2010). If this physiological function by the ciliary body is inhibited by some etiology such as virus infection, the aqueous circulation will not function smoothly. As a result, the lens will be subjected to physiological dysfunction resulting in a disorder such as a cataract. Since the ciliary body has such an important physiological function, we suggest strongly that virus infection of the ciliary body might play an important role in cataractogenesis.

In conclusion, we reported here the pathological features of the congenital rubella virus infection in human fetuses. The virus infection occurred via the systemic organs of the human fetuses. To the best of our knowledge, this study is the first report to demonstrate that the rubella virus infects via the systemic organs in the human body. Importantly, we identified that the virus infected the ciliary body and this could play an important role in cataractogenesis. We hope that our report presented here will help to elucidate the mechanism of CRS occurrence in fetuses/newborns caused by congenital RV infection during pregnancy and to control rubella in order to improve maternal and child health worldwide.

## Funding

None.

## Competing Interests

None.

## Figures and Tables

**Table 1:** Detection of RV-specific protein and RV RNA in multiple organs from fetuses with congenital rubella infection.

Case no.	Fetus age/sex	Mother infected at	RV Ag1/RV RNA2 detection in multiple organs							
Placenta	CNS3	Lung	Heart	Liver	Kidney	Spleen	Eye
1	23 weeks/M	Week 5 of gestation	NT4/+	+/+	+/+	+/+	+/+	+/+	+/+	+/+
2	22 weeks/F	Week 6 of gestation	NT/+	+/+	+/+	+/+	+/+	+/+	NT/+	+/+
3	13 weeks/M	Week 6 of gestation	+/+	+/+	NT/+	+/+	+/+	+/+	NT/+	+/+

1. Determined by immunohistochemistry for RV capsid antigen.

2. Determined by nested RT-PCR for negative-strand RV RNA.

3. CNS = central nervous system.

4. NT = not tested due to tissue fragments are lost.
